# LINC00511 contributes to glioblastoma tumorigenesis and epithelial‐mesenchymal transition via LINC00511/miR‐524‐5p/YB1/ZEB1 positive feedback loop

**DOI:** 10.1111/jcmm.14829

**Published:** 2019-12-19

**Authors:** Xiaoliu Du, Yiming Tu, Shuang Liu, Pengzhan Zhao, Zhongyuan Bao, Chong Li, Jinhao Li, Minhong Pan, Jing Ji

**Affiliations:** ^1^ Department of Pathology The First Affiliated Hospital of Nanjing Medical University Nanjing Jiangsu China; ^2^ Department of Neurosurgery The First Affiliated Hospital of Nanjing Medical University Nanjing Jiangsu China; ^3^ Department of Pediatrics The First Affiliated Hospital of Nanjing Medical University Nanjing Jiangsu China

**Keywords:** epithelial‐mesenchymal transition, glioblastoma, LINC00511, tumorigenesis, ZEB1

## Abstract

Tumour invasion is closely related to the prognosis and recurrence of glioblastoma multiforme and partially attributes to epithelial‐mesenchymal transition. Long intergenic non‐coding RNA 00511 (LINC00511) plays a pivotal role in tumour; however, the role of LINC00511 in GBM, especially in the epigenetic molecular regulation mechanism of EMT, is still unclear. Here, we found that LINC00511 was up‐regulated in GBM tissues and relatively high LINC00511 expression predicted poorer prognosis. Moreover, ectopic LINC00511 enhanced GBM cells proliferation, EMT, migration and invasion, whereas LINC00511 knockdown had the opposite effects. Mechanistically, we confirmed that ZEB1 acted as a transcription factor for LINC00511 in GBM cells. Subsequently, we found that LINC00511 served as a competing endogenous RNA that sponged miR‐524‐5p to indirectly regulate YB1, whereas, up‐regulated YB1 promoted ZEB1 expression, which inversely facilitated LINC00511 expression. Finally, orthotopic xenograft models were performed to further demonstrate the LINC00511 on GBM tumorigenesis. This study demonstrates that a LINC00511/miR‐524‐5p/YB1/ZEB1 positive feedback loop provides potential therapeutic targets for GBM progression.

## 1 INTRODUCTION

Glioblastoma multiforme (GBM) is the most common and aggressive primary brain tumour^1^, ^2^ and has a poor prognosis^3^, ^4^, ^5^ despite the fact that advanced treatment methods such as surgical resection followed by neoadjuvant chemotherapy and radiotherapy have been implemented. Studies have implicated that the poor prognosis of GBM is largely attributable to enhanced epithelial‐mesenchymal transition (EMT), which promotes tumour cell invasion.^6^, ^7^ Because of the presence of invasion, it is impossible to perform a complete resection with surgery in patients with GBM, which directly accounts for recurrence and dissemination.^8^, ^9^ Therefore, we should search for a new prognostic biomarker and/or therapeutic target for patients with GBM.

EMT is a complicated progress that promotes stemness and invasion^10^, ^11^ and is accompanied by the up‐regulation of mesenchymal markers (Vimentin, N‐cadherin and Fibronectin) and down‐regulation of epithelial markers (E‐cadherin, Catenin). Meanwhile, the EMT process is also regulated by the transcription factors Slug, Snail, Zinc finger E‐box binding homeobox 1 (ZEB1) and Twist.^12^, ^13^, ^14^, ^15^ Among which, ZEB1 plays an indispensable role in the activation of EMT. Studies have reported that the long non‐coding RNA (lncRNA) RP11 promotes colorectal cancer cells dissemination by ZEB1 overexpression, whereas lncRNA HCCL5 could be activated by ZEB1 to promote hepatocellular carcinoma malignancy.^13^, ^14^ It has also been reported that ZEB1 is regulated by some microRNAs (miRNAs).^16^, ^17^

Non‐coding RNAs (ncRNAs) are transcribed genes that are not further translated into proteins and include lncRNAs, circular RNAs (circRNAs) and miRNAs, which perform vital roles in human cancers.^18^, ^19^ Among these, lncRNAs are a class of ncRNAs of more than 200 bp that have been demonstrated to be closely related to cancer progression, including tumorigenesis,^20^ stemness,^21^ apoptosis,^22^ chemoresistance^23^ and EMT.^24^ LINC00511, a 2265 bp ncRNA located on chromosome 17q24.3, has been demonstrated in several tumours. For examples, LINC00511 promotes breast cancer tumorigenesis and stemness.^25^ It has also been reported that LINC00511 promotes proliferation in pancreatic ductal adenocarcinoma,^26^ ovarian cancer^27^ and glioma.^28^ However, the molecular mechanisms of LINC00511 in EMT are still unclear.

This study identified that LINC00511 was up‐regulated in GBM, where it was associated with a worse prognosis, and that silencing LINC00511 markedly inhibited proliferation and EMT of GBM cells. Mechanistically, we found that a LINC00511/miR‐524‐5p/YB1/ZEB1 positive feedback loop could detailedly illustrate the molecular mechanism of LINC00511 in GBM.

## 2 MATERIALS AND METHODS

### 2.1 Clinical specimens

We obtained 36 GBM tissues and 8 non‐tumour brain tissues (NBT) from the Department of Neurosurgery, The First Affiliated Hospital of Nanjing Medical University between 2013 and 2018. The GBM specimens were collected from patients diagnosed with GBM by histopathological evaluation. NBT were collected from patients undergoing surgery after physical brain injury. The clinicopathological data of patients with GBM are shown in Table S1. Our study was approved by Research Ethics Committee of Nanjing Medical University, and informed consent for the usage of specimens was received from all patients.

### 2.2 Cell culture

We purchased four human GBM cell lines (U87, LN229, U251 and A172) and human embryonic kidney (HEK) 293T cells from the Chinese Academy of Sciences Cell Bank. N3 primary culture cells were donated from Tian Tan Hospital, and a normal human astrocyte line (NHA) was purchased from Sciencell Research Laboratories. GBM cells and HEK 293T cells were cultured in Dulbecco's Modified Eagle Medium (DMEM), and NHA cells were cultured in astrocyte medium. Both medium were mixed with 10% foetal bovine serum (FBS). All cells were cultured at 37°C with 5% CO_2_.

### 2.3 RNA extraction and qRT‐PCR

Total RNA was extracted from GBM tissues and cell lines using TRIZOL reagent (Invitrogen). cDNA was produced using the PrimeScrip‐RT Reagent Kit (Takara). qRT‐PCR was performed with the SYBR Green PCR kit (Takara) on an ABI 7500 real‐time PCR system (Applied Biosystems). The results were normalized to GAPDH expression and analysed using the 2^−ΔΔCt^ method. The specific primers (Ribobio) are listed in Table S2.

### 2.4 Transfection and transduction

To establish stable cells for tumorigenicity assays, lentiviruses carrying si‐LINC00511 (sh‐LINC00511) or vectors were purchased from Genechem. Stable cells were established by lentiviral transduction and puromycin selection as previous study described.^7^ For transient transfection, small interfering RNAs (siRNAs) and plasmids were purchased from Ribobio and transfected into N3 and U251 cells using Lipofectamine 3000 (Invitrogen). The exact sequences are presented in Table S3. miR‐524‐5p inhibitor and mimics were also purchased from Ribobio and transfected into GBM cells using Lipofectamine RNAiMAX Reagent (Life Technologies).

### 2.5 Cell proliferation assays

Cell vitality was detected by the Cell Counting Kit‐8 (CCK‐8) assay kit (DOJINDO). Briefly, transfected cells were seeded into 96‐well plates and incubated with CCK‐8 reagent every 24 hours according to manufacturer's instructions. For colony formation assay, cells were cultured in 6‐well plates, and after approximately 14 days, the cells were fixed with methanol and stained with 0.1% crystal violet. Visible colonies were photographed.

### 2.6 Ethynyl deoxyuridine (EdU) analysis

Cells were seeded in 96‐well plates and processed with the EdU labeling/detection kit (Ribobio) following the manufacturer's protocol. Positive cells were detected by fluorescence microscopy (Olympus). We chose five random fields from each well to evaluate the percentage of EdU‐positive cells.

### 2.7 Immunohistochemical (IHC) and Western blot analysis

Tissues for IHC were collected from orthotopic xenograft tumour models. Total proteins were extracted from GBM tissues and cell lines using RIPA extraction reagent (Beyotime) supplemented with proteinase inhibitor (Beyotime) as previously reported.^7^, ^29^ The antibodies used in this study were as follows: Vimentin (#5741), N‐cadherin (#13116), E‐cadherin (#3195), YB1 (#8475), CDK4 (#12790), Snail (#3879), Actin (#3700), GAPDH (#5174) (Cell Signaling Technology); ZEB1 (ab124512) and Slug (ab180714) (Abcam); and p21 (10355‐1‐AP) and Cyclin D1 (60186‐1‐Ig) (Proteintech).

### 2.8 Cell migration and invasion assays

Wound healing, 3D spheroid invasion and transwell assays were performed as previously described^30^ to investigate the migration and invasion ability of GBM cells.

### 2.9 Flow cytometric analysis

Cells were fixed in 75% ethanol overnight and then stained with propidium iodide (PI) using BD Pharmingen™ PI/RNase Staining Buffer (BD, Biosciences) according to the manufacturer's protocol. The percentages of cells in G0/G1, S and G2/M phase were obtained and analysed using Wincycle‐32 bit.

### 2.10 Fluorescence in situ hybridization (FISH) and subcellular fractionation

Tissues or cells were fixed in 4% formaldehyde for 15 minutes, and 0.5% TritonX‐100 was used for permeabilization at 4°C for 15 minutes. Then, digoxigenin (DIG)‐labelled LINC00511 probe or control probe incubated with the cells at 55°C overnight. DAPI was performed to counterstain nuclei. A confocal laser scanning microscope (Carl Zeiss) was used to obtain images. The isolation and purification of nuclear and cytoplasmic RNA were done using the PARIS Kit (Life Technologies) following manufacturer's protocol.

### 2.11 RNA immunoprecipitation (RIP)

The EZMagna RIP Kit (Millipore) was used for RIP according to the manufacturer's protocol. Cells were lysed and incubated at 4°C with protein A magnetic beads, which could be conjugated with Ago2 (Millipore), whereas IgG alone (Millipore) served as the negative control. Then, the purified RNA products were tested by qRT‐PCR to prove the presence of LINCOO511 or miR‐524‐5p.

### 2.12 Dual‐luciferase gene reporter assay

Dual‐Luciferase Kit (Promega) was applied for luciferase assay as previously reported.^30^

### 2.13 Chromatin immunoprecipitation (ChIP)

The ChIP assay was performed with the Simple Chip Enzymatic Chromatin IP Kit (Cell Signaling Technology) according to manufacturer's instructions. Briefly, cells were incubated with specific antibody, and then, the purified immunoprecipitated DNA was obtained and detected by qRT‐PCR or RT‐PCR using specific primers as shown in Table S4.

### 2.14 The orthotopic xenograft model

Male BALB/c nude mice (6‐week‐old) were supplied by Beijing Vital River Laboratory Animal Technology Co. Ltd. Luciferase‐labelled U251 cells transduced with sh‐LIN00511 or empty vector were prepared (10 mice per group) and then inoculated into nude mice. The resulting tumours were analysed by bioluminescence imaging.

### 2.15 Statistical analysis

All experiments were performed thrice independently. Data were analysed by SPSS 20.0 software and graphed by GraphPad Prism 5.0 Software (La Jolla, CA, USA). Data were shown as mean ± standard error of mean (SEM) for Student's *t* test or one‐way ANOVA. The relationship between two genes was explored using Pearson' correlation method. Survival analysis was performed by the Kaplan‐Meier method. *P* < .05 was considered statistically significant.

## 3 RESULTS

### 3.1 LINC00511 is up‐regulated in GBM tissues and cells and associated with poor prognosis

**Figure 1:**
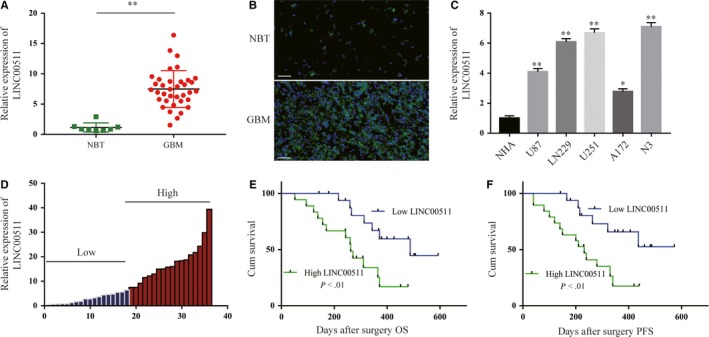
LINC00511 is up‐regulated in GBM tissues and cells and associated with poor prognosis. A, Relative expression of LINC00511 in GBM tissues (n = 36) compared with NBT (n = 8) was detected by qRT‐PCR. B, FISH was performed in GBM tissues and NBT. Scale bar, 50 μm. C, LINC00511 expression in NHA and GBM cell lines was analysed by qRT‐PCR. D, Two groups were arranged in GBM patients according to the mean value. E and F, Kaplan‐Meier overall survival and disease‐free survival curves depending on LINC00511 expression levels. Error bars indicate mean ± standard error of the mean (SEM). *P < .05, **P < .01

Through an extensive article readings and search of bioinformatics databases, LINC00511 caught our attention. Based on these preliminary results, qRT‐PCR analysis of LINC00511 expression in 36 GBM tissues and 8 NBT showed that LINC00511 was markedly increased in GBM tissues compared with NBT (Figure 1A). FISH was performed in human tissues as shown in Figure 1B. Additionally, LINC00511 expression was found to be significantly increased in human GBM cell lines and revealed a much higher expression in N3 and U251 cells (Figure 1C). To further explore the relationship between LINC00511 expression and clinicopathological indexes in patients with GBM, we divided the 36 patients with GBM into two groups of those with relatively high or low LINC00511 expression according to the median level of LINC00511 (n = 18 ≥ median; n = 18 ≤ median) (Figure 1D and Table S1). The high LINC00511 expression group was more positively correlated with larger tumour size (*P* = .026), wild‐type IDH1/2 (*P* = .043), recurrence (*P* = .011) and poor prognosis than the low LINC00511 expression group, however, not with other features such as gender (*P* = .157) and age (*P* = .7). Kaplan‐Meier analysis further evaluates the correlation between LNC00511 expression and the prognosis of patients with GBM after surgery (Figure 1E,F). Taken together, these data show that LINC00511 could be a novel biomarker for diagnosis and prognosis.

### 3.2 LINC00511 enhances GBM cells proliferation

**Figure 2:**
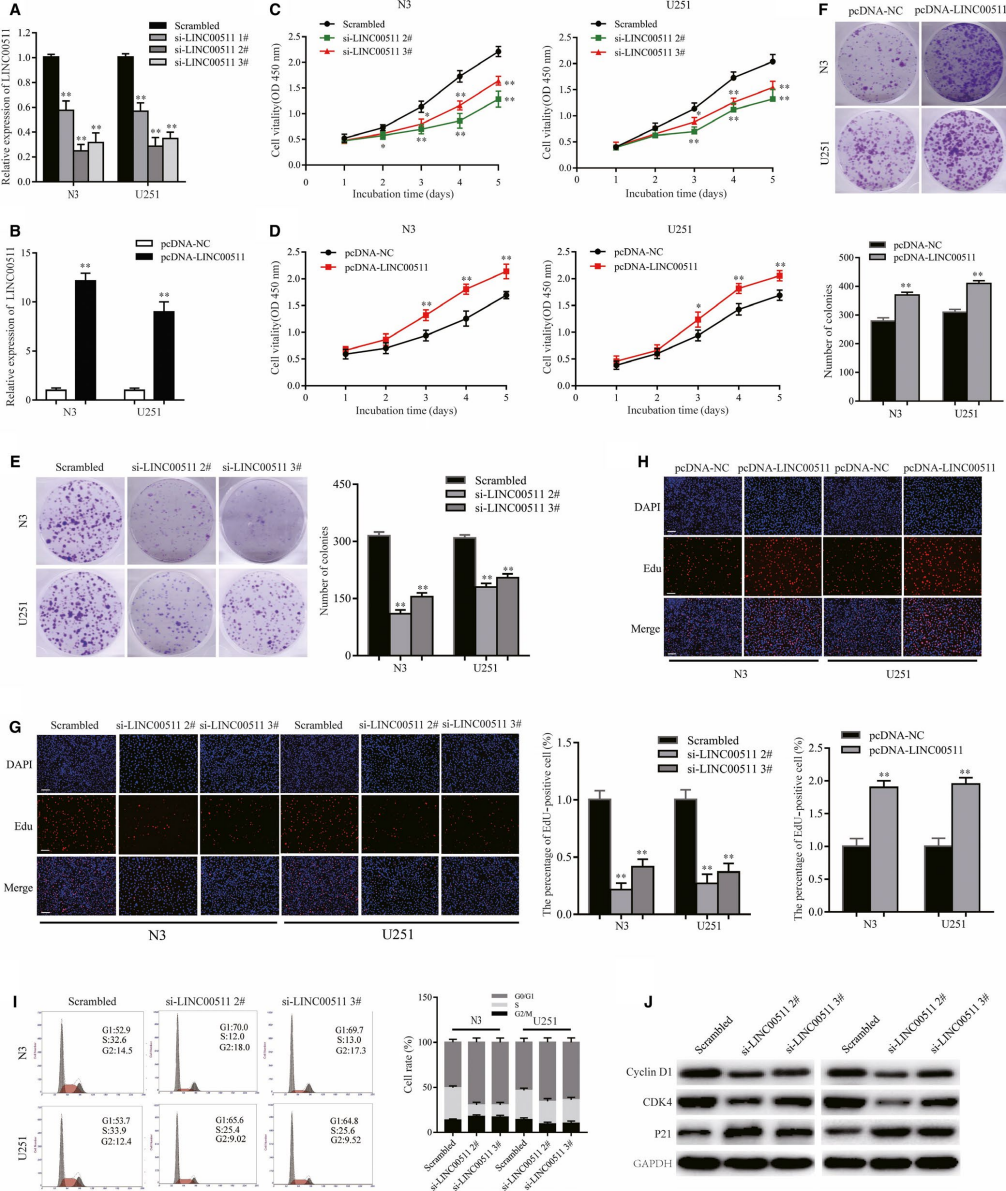
Effect of LINC00511 on GBM cells proliferation and cell cycle in vitro. A and B, LINC00511 expression in N3 and U251 cells transfected with control, si‐LINC00511 1#, 2#, 3# and pcDNA‐LINC00511 was detected by qRT‐PCR analysis. C and D, The viability of N3 and U251 cells transfected with si‐LINC00511 or pcDNA‐LINC00511 was performed by CCK8 assay. E and F, colony formation assay was used to detect the proliferation of si‐LINC00511‐transfected and pcDNA‐LINC00511‐transfected GBM cells. G and H, The proliferation of GBM‐transfected cells was examined by EdU staining assay. Scale bar, 100 μm. I, Flow cytometry was performed to identify the proportion of si‐LINC00511‐transfected cells in G1, S or G2 phase, respectively. J, Western blot analysis of cycle‐related proteins in N3 and U251 cells transfected with si‐LINC00511 or scrambled. GAPDH protein was applied as an internal control. The data represent the mean of three independent experiments ± SEM. *P < .05, **P < .01

To further investigate the function of LINC00511 in GBM, we designed three independent siRNAs and transfected them into N3 and U251 cells. qRT‐PCR analysis of LINC00511 expression indicated that si‐LINC00511 2# and 3# had higher interference efficiency and were chose for the following experiments (Figure 2A). Meanwhile, overexpression plasmid (pcDNA3.1‐LINC00511) and empty pcDNA3.1 vector (pcDNA‐NC) were transfected into cells as well (Figure 2B).

The CCK8 assay indicated that LINC00511 knockdown significantly decreased the proliferation of N3 and U251 cells (Figure 2C). In contrast, LINC00511 overexpression strongly increased cell proliferation (Figure 2D). Meanwhile, colony formation assay showed that silencing LINC00511 inhibited clonogenic survival in both N3 and U251 cells (Figure 2E). Colony formation was markedly promoted by enhancing LINC00511 levels (Figure 2F). Similarly, EdU immunostaining revealed similar results (Figure 2G,H).

Cell cycle arrest and apoptosis are important factors for cancer cell proliferation. Flow cytometric assay showed that LINC00511 knockdown induced cell cycle arrest (Figure 2I). Importantly, more cells remained in G1/G0 phase compared with controls. However, there were no significant differences in the level of apoptosis (data not shown). Western blot analysis revealed that levels of G1/S phase‐related proteins Cyclin D1 and CDK4 were significantly decreased, whereas p21 was increased after transfecting N3 and U251 cells with si‐LINC00511 (Figure 2J). These findings suggest that LINC00511 behaves as an oncogene in GBM cells.

### 3.3 LINC00511 promotes migration and invasion by enhancing EMT in GBM cells

**Figure 3:**
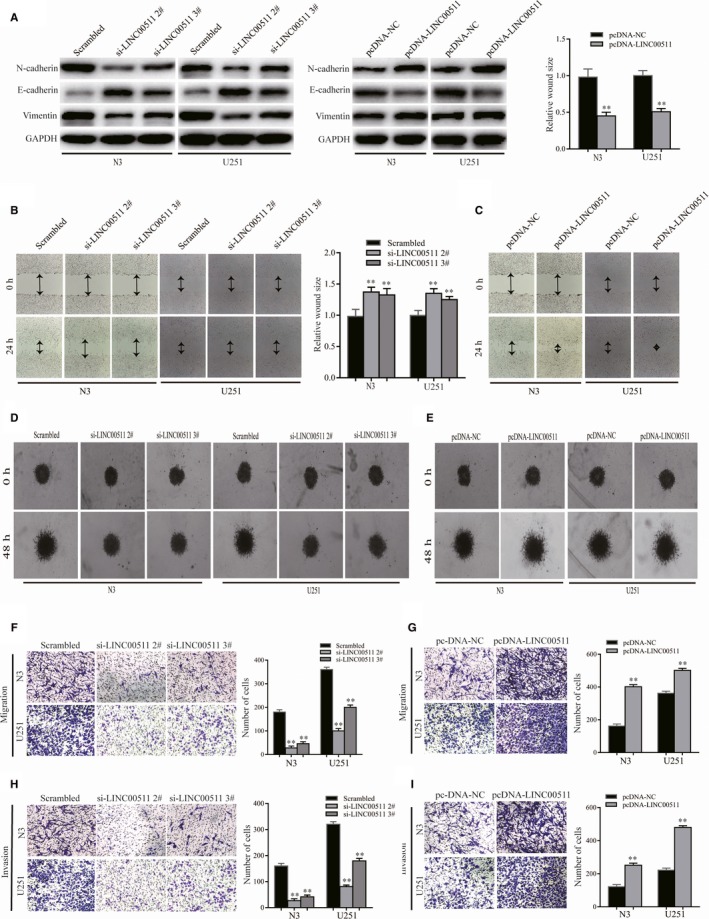
Effect of LINC00511 on EMT, migration and invasion in GBM cells in vitro. A, Western blotting was performed to detect the expression of EMT‐related proteins N‐cadherin, E‐cadherin and Vimentin in N3 and U251 cells transfected with si‐LINC00511 or pcDNA‐LINC00511 compared with respective control. B‐E, Wound healing and 3D spheroid invasion assays were performed to evaluate the ability of migration or invasion. F‐I, Transwell assay was arranged to detect the changes of GBM cells transfected with si‐LINC00511 or pcDNA‐LINC00511 in migration and invasion. Values represent the mean ± SEM in three independent experiments. *P < .05, **P < .01

To explore whether changes in LINC00511 expression affect GBM cells EMT in vitro, we analysed the expression of proteins associated with EMT. The results showed that down‐regulating LINC00511 in both N3 and U251 cells decreased expression of the mesenchymal markers Vimentin and N‐cadherin but increased E‐cadherin expression. Conversely, up‐regulating LINC00511 had the opposite effect (Figure 3A).

To further explore whether the influence of LINC00511 on migration and invasion is induced by EMT, the wound healing and 3D spheroid invasion assays were performed in N3 and U251 cells transfected with si‐LINC00511 or pcDNA‐LINC00511 (Figure 3B‐E). Transwell assay also showed similar results (Figure 3F‐I). Taken together, these findings suggest that LINC00511 accelerates GBM cells migration and invasion *via* enhancing EMT.

### 3.4 ZEB1 regulates LINC00511 at the transcription level

**Figure 4:**
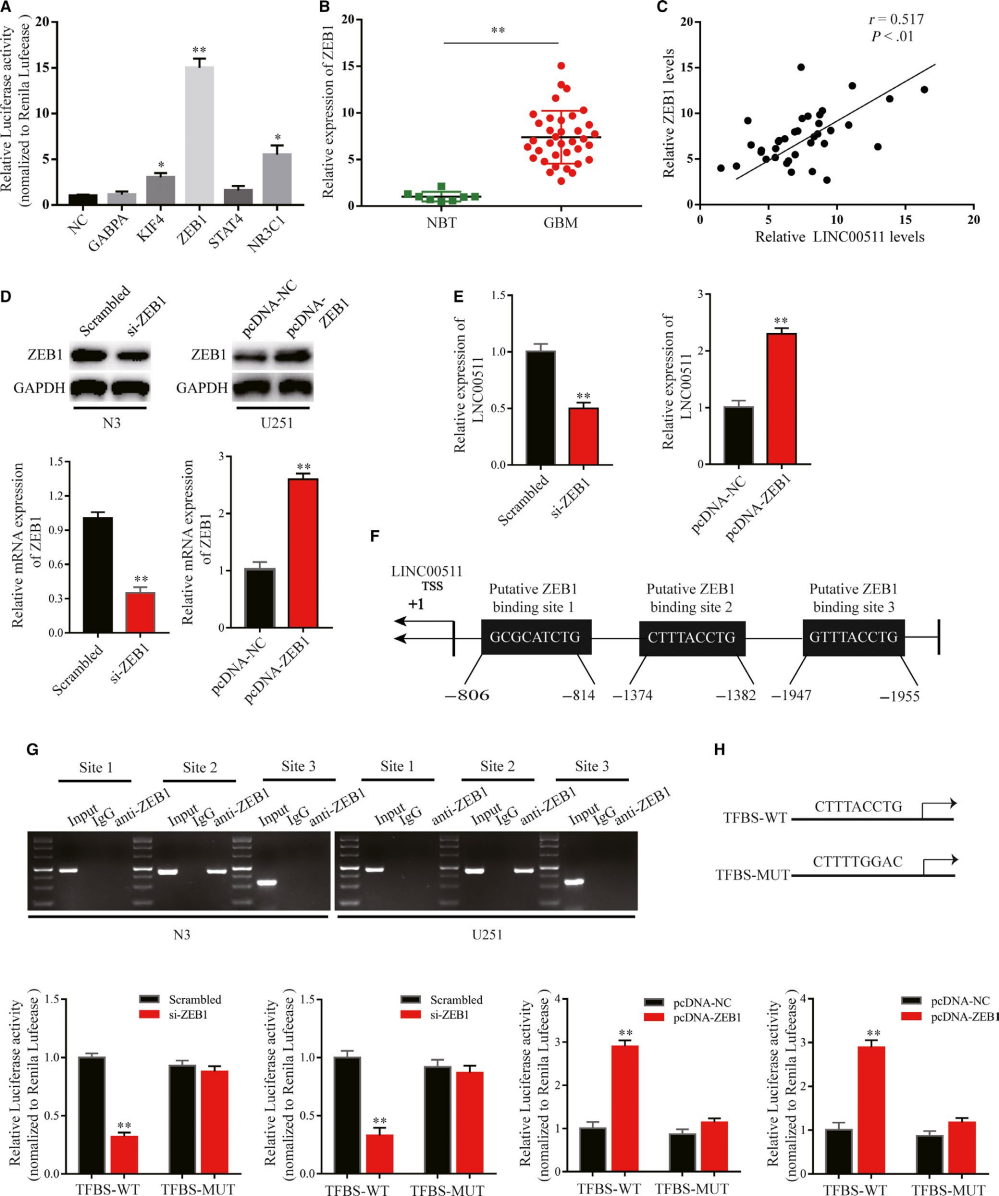
ZEB1 could positively regulate LINC00511 at the transcription level. A, The luciferase activity in HEK‐293T cells co‐transfected plasmids containing LINC00511 with plasmids containing transcription factors. B, qRT‐PCR analysis of ZEB1 expression in GBM tissues (n = 36) and NBT (n = 8). C, Pearson's correlation analysis of the correlation between LINC00511 and ZEB1. D, ZEB1 expression in si‐ZEB1‐transfected or pcDNA‐ZEB1‐transfected N3 and U251 cells at protein and mRNA levels. E, Relative expression of LINC00511 in si‐ZEB1‐transfected or pcDNA‐ZEB1 N3 and U251 cells. F, Schematic diagram revealing the human LINC00511 promoter region and ZEB1 potential binding site. G, The relative enrichment of ZEB1 on promoter region of LINC00511 was detected by ChIP assay. H, Schematic view of ZEB1 putative targeting site in wild‐type and mutant TFBS (up). Luciferase reporter assay was performed in N3 and U251 cells co‐transfected si‐ZEB1 or pcDNA‐ZEB1 with wild‐type or mutated TFBS (down). Values represent the mean ± SEM of three independent experiments. *P < .05, **P < .01

Many studies have found that differential expression of lncRNAs can be attributed to upstream transcription inducers.^31^ To understand the molecular regulatory mechanism of LINC00511 in GBM cells, highly likely transcription factors were predicted using the JASPAR database (http://jaspar.binf.ku.dk/), and the five transcription factors with the highest prediction scores were selected for luciferase assay. Briefly, a plasmid containing the LINC00511 promoter region was transfected into HEK‐293T cells with plasmids including transcription factors or control sequence. Then, we found that ZEB1 had the strongest luciferase induction among them (Figure 4A). ZEB1 as a key inducer enhances EMT in GBM to promote migration and invasion^7^, ^32^; thus, we considered it the best candidate for subsequent studies.

Firstly, we detected ZEB1 expression in GBM tissues and NBT, and analysed the correlation between LINC00511 and ZEB1 (Figure 4B,C). To further explore our hypothesis, we inhibited or enhanced ZEB1 expression in N3 and U251 cells by transfection with si‐ZEB1 or pcDNA‐ZEB1 (Figure 4D). The results showed that LINC00511 expression was reduced or increased according to ZEB1 levels (Figure 4E). Nextly, by scanning the promoter region of LINC00511 with the JASPER database, three potential binding sites were found (Figure 4F). The ChIP assay was used to evaluate the most likely binding site between ZEB1 and the LINC00511 promoter region. qRT‐PCR results showed that ZEB1 bound to LINC00511 only at Site 2, the −1382 to −1374 bp region (Figure 4G). Additionally, luciferase assay further suggested that ZEB1 as a transcription factor positively regulates LINC00511 in GBM cells (Figure 4H).

### 3.5 LINC00511 acts as a competing endogenous (ce) RNA by sponging miR‐524‐5p

**Figure 5:**
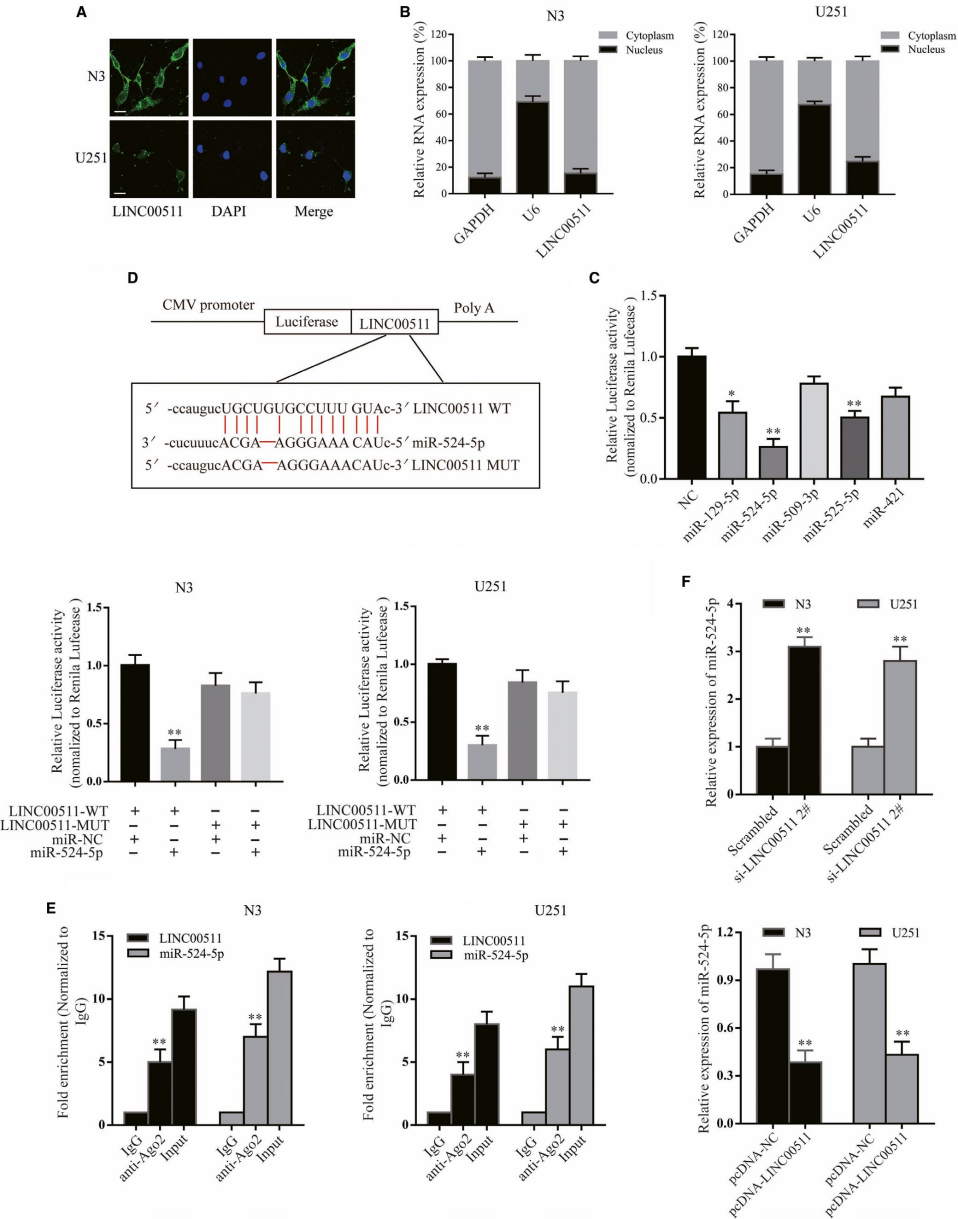
The relationship between LINC00511 and miR‐524‐5p. A and B, The location of LINC00511 in the nucleus and cytoplasm was detected in N3 and U251 cells by FISH and subcellular fractionation assays. Scale bar, 20 μm. C, The luciferase activity in HEK‐293T cells co‐transfected plasmids containing LINC00511 with 5 various miRNA‐coding plasmids. D, The binding site of miR‐524‐5p and LINC00511 was verified by luciferase reporter assay. E, RIP assay was performed with IgG or Ago2 antibodies in N3 and U251 cells, and the coprecipitated RNA was subjected to qRT‐PCR for LINC00511 and miR‐524‐5p. F, qRT‐PCR analysis of miR‐524‐5p in N3 and U251 cells transfected with si‐LINC00511 (up) or pcDNA‐LINC00511 (down). Data represent the mean ± SEM of three independent experiments. *P < .05, **P < .01

As mentioned before, we detected the effect of LINC00511 on EMT‐related proteins. Then, we investigated the influence of LINC00511 on EMT inducers (Slug, ZEB1 and Twist). Western blot analysis showed that ZEB1 expression was significantly decreased by inhibiting LINC00511 in N3 cells at the protein and mRNA levels; however, the EMT inducers Slug and Twist were unchanged (Figure S1A). Similar to LINC00511 overexpression, ZEB1 was markedly increased in U251 cells at the protein and mRNA levels (Figure S1B), suggesting that LINC00511 can regulate the expression of ZEB1 both at the mRNA and protein levels. However, the exact regulatory mechanism was still unclear. To further explore the possible mechanism through which LINC00511 could promote GBM progression by regulating ZEB1, FISH and subcellular fractionation were performed to examine its subcellular localization, which determine its function.^33^ Interestingly, the results of both showed that LINC00511 was primarily localized in the cytoplasm rather than nucleus of GBM cells (Figure 5A,B). Studies have reported that cytoplasmic lncRNAs can adjust target gene levels by acting as a ceRNAs or directly combining with RNA‐binding proteins,^34^, ^35^ suggesting that LINC00511 might function as a ceRNA to regulate target genes. Thus, StarBase v2.0 software was used to predict potential target miRNAs. Taking all factors into consideration, miR‐129‐5p, miR‐524‐5p, miR‐509‐3p, miR‐525‐5p and miR‐421 were chosen for further screening using luciferase assay. The results revealed that miR‐524‐5p had the most suppression ability of LINC00511‐driven luciferase activity in HEK‐293T cells (Figure 5C). We then constructed LINC00511 luciferase reporter vectors including wild‐type and a construct in which the putative binding sites for miR‐524‐5p were mutated for further demonstration. As anticipated, when wild‐type LINC00511, not mutated construct, was co‐transfected with miR‐524‐5p there was significantly reduced luciferase activity in both cell lines (Figure 5D). Additionally, a previous study showed that Ago2 is a component of the RNA‐induced silencing complex (RISC) that contributes to miRNA‐mediated repression of mRNAs.^36^ To determine whether LINC00511 and miR‐524‐5p could bind to RISC, the RIP assay was performed and showed that LINC00511 and miR‐524‐5p could directly bind Ago2 (Figure 5E). In GBM cells (N3, U251), miR‐524‐5p expression was reduced (Figure S1C). Moreover, miR‐524‐5p expression was increased following LINC00511 reduction (si‐LINC00511 2# was used for rescue assay) and decreased following LINC00511 overexpression (Figure 5F). Taken together, miR‐524‐5p is the best candidate for LINC00511.

### 3.6 LINC00511 activity is partially moderated by negatively regulating miR‐524‐5p

To further explore the regulatory mechanism between LINC00511 and miR‐524‐5p, the expression of miR‐524‐5p in GBM tissues were detected by qRT‐PCR, and the correlation between LINC00511 and miR‐524‐5p was analysed (Figure S1D). Next, we performed rescue experiments to revalidate the regulatory mechanism. First, the CCK8 assay was performed to examine cell viability. The results showed that silencing LINC00511 markedly reduced cell viability; however, this was rescued by co‐transfection with a miR‐524‐5p inhibitor (Figure S2A). Next, we explored the expression of EMT‐related markers in N3 and U251 cells co‐transfected with si‐LINC00511 and miR‐524‐5p inhibitor (Figure S2B). Colony formation and EdU assays were also performed to verify our hypothesis (Figure S2C,D). Additionally, wound healing and transwell assays showed that the inhibition of migration and invasion following transfection with si‐LINC00511 was rescued by co‐transfecting the miR‐524‐5p inhibitor (Figure S2E‐G). These results illustrate that LINC00511 enhances proliferation, EMT, migration and invasion in GBM cells partially by suppressing miR‐524‐5p activity.

### 3.7 LINC00511 acts as a ceRNA that indirectly regulates YB1 by sponging miR‐524‐5p

**Figure 6:**
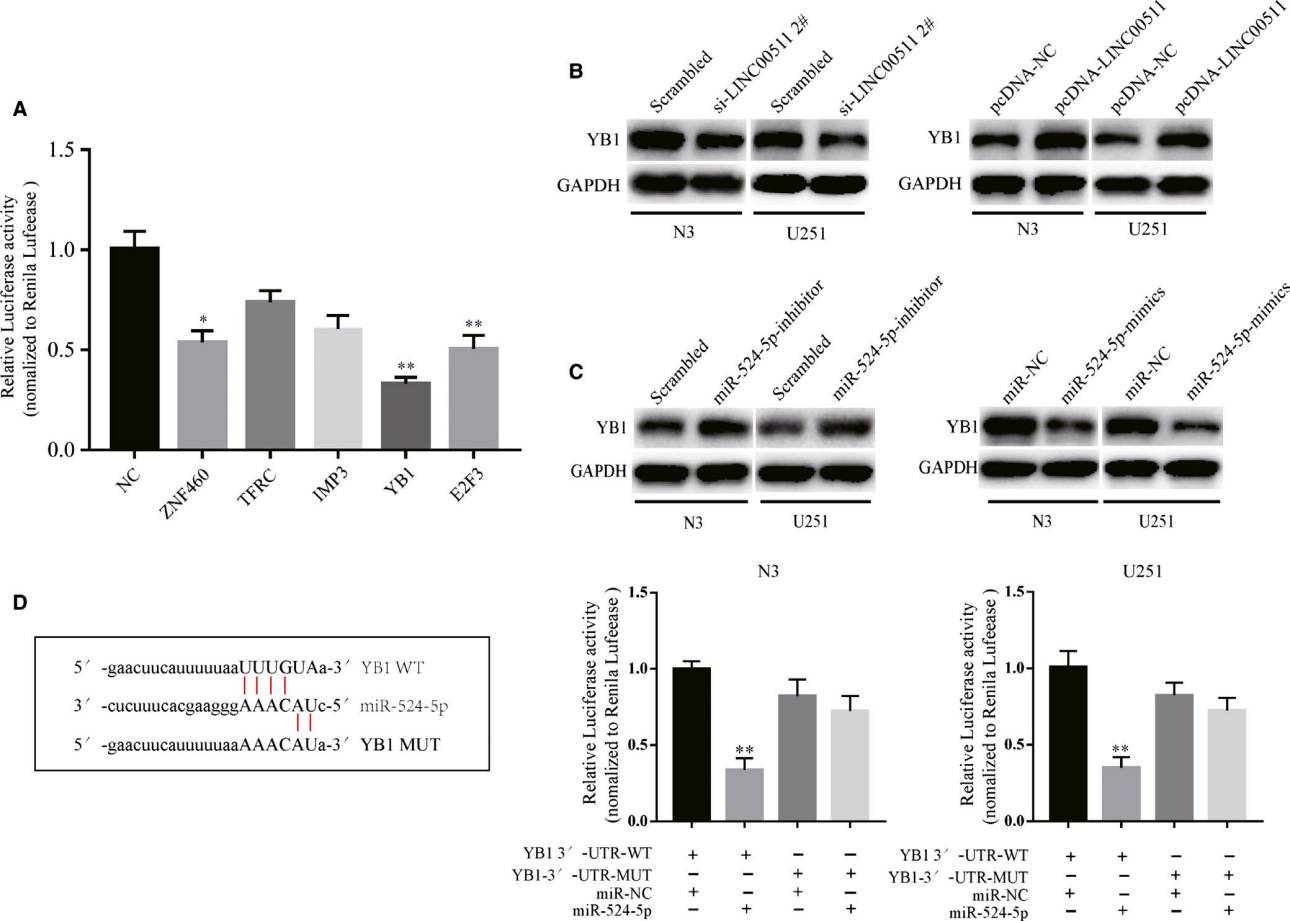
The relationship between miR‐524‐5p and YB1. A, The luciferase activity in HEK‐293T cells co‐transfected plasmids containing LINC00511 with plasmids containing potential targeting genes. B, The expression of YB1 in si‐LINC00511‐transfected or pcDNA‐LINC00511‐transfected N3 and U251 cells. C, YB1 expression in miR‐524‐5p‐inhibitor‐transfected or miR‐524‐5p‐mimic‐transfected GBM cells. D, Schematic illustration of miR‐524‐5p binding site in YB1 and the site mutagenesis (left). The luciferase reporter plasmids containing wild‐type or mutant type YB1 was co‐transfected into N3 and U251 cells with miR‐524‐5p or empty vector (right). Values represent the mean ± SEM of three independent experiments. *P < .05, **P < .01

The function of ceRNA networks involves lncRNAs indirectly regulating downstream genes by targeting miRNAs. Therefore, we searched for target genes using TargetScan and StarBase v2.0. Then, we analysed the luciferase activity of the five most likely target genes. The data revealed that YB1 had the most correlation with miR‐524‐5p (Figure 6A). Next, we detected changes in the protein level of YB1 in N3 and U251 cells transfected with si‐LINC00511 or pcDNA‐LINC00511 (Figure 6B). Meanwhile, knockdown or overexpression of miR‐524‐5p also influenced YB1 expression in GBM cells (Figure 6C). Additionally, the luciferase assay demonstrated that GBM cells co‐transfected with YB1‐WT and miR‐524‐5p markedly inhibited the activity of luciferase compared with the other groups (Figure 6D). Taken together, these suggest that LINC00511 can indirectly regulate YB1 by sponging miR‐524‐5p.

### 3.8 LINC00511 indirectly promotes ZEB1 expression by sponging miR‐524‐5p to target YB1

As the literature reported, YB1 has a potential binding site in the ZEB1 promoter region, and YB1 acts as a transcription factor that positively regulates ZEB1 in pancreatic cancer.^37^ Therefore, we tested whether YB1 had the same effect on ZEB1 in GBM cells. Western blot assay showed that YB1 knockdown decreased ZEB1 protein levels, whereas overexpressing YB1 increased ZEB1 expression (Figure S3A). Next, we scanned the binding site using the catRAPID database (Figure S3B).^37^ ChIP assay revealed that the ZEB1 promoter was enriched for the YB1‐bound complex (Figure S3C).

To further investigate the regulatory function of LINC00511 on ZEB1, we co‐transfected si‐LINC00511 and pcDNA‐YB1 with wild‐type or mutated ZEB1 promoter. The luciferase assay showed that LINC00511 knockdown inhibited luciferase activity in ZEB1‐WT, but not ZEB1‐MUT. However, the result was significantly reversed *via* co‐transfection of pcDNA‐YB1 (Figure S3D). Western blot analysis also revealed that si‐LINC00511‐mediated reduction of ZEB1 expression was partially rescued by co‐transfection with pcDNA‐YB1 (Figure S3E,F). Analyses of mRNA levels showed the same results (Figure S3E,F). We then examined the expression of EMT‐related proteins in si‐LINC00511 and pcDNA‐ZEB1 co‐transfected N3 and U251 cells (Figure S3G). Transwell assay was also performed to demonstrate the relationship between LINC00511 and ZEB1 (Figure S3H,I). Taken together, we conclude that LINC00511 promotes EMT in GBM cells through the LINC00511/miR‐524‐5p/YB1/ZEB1 positive feedback loop.

### 3.9 LINC00511 accelerates GBM tumorigenesis in vivo

**Figure 7:**
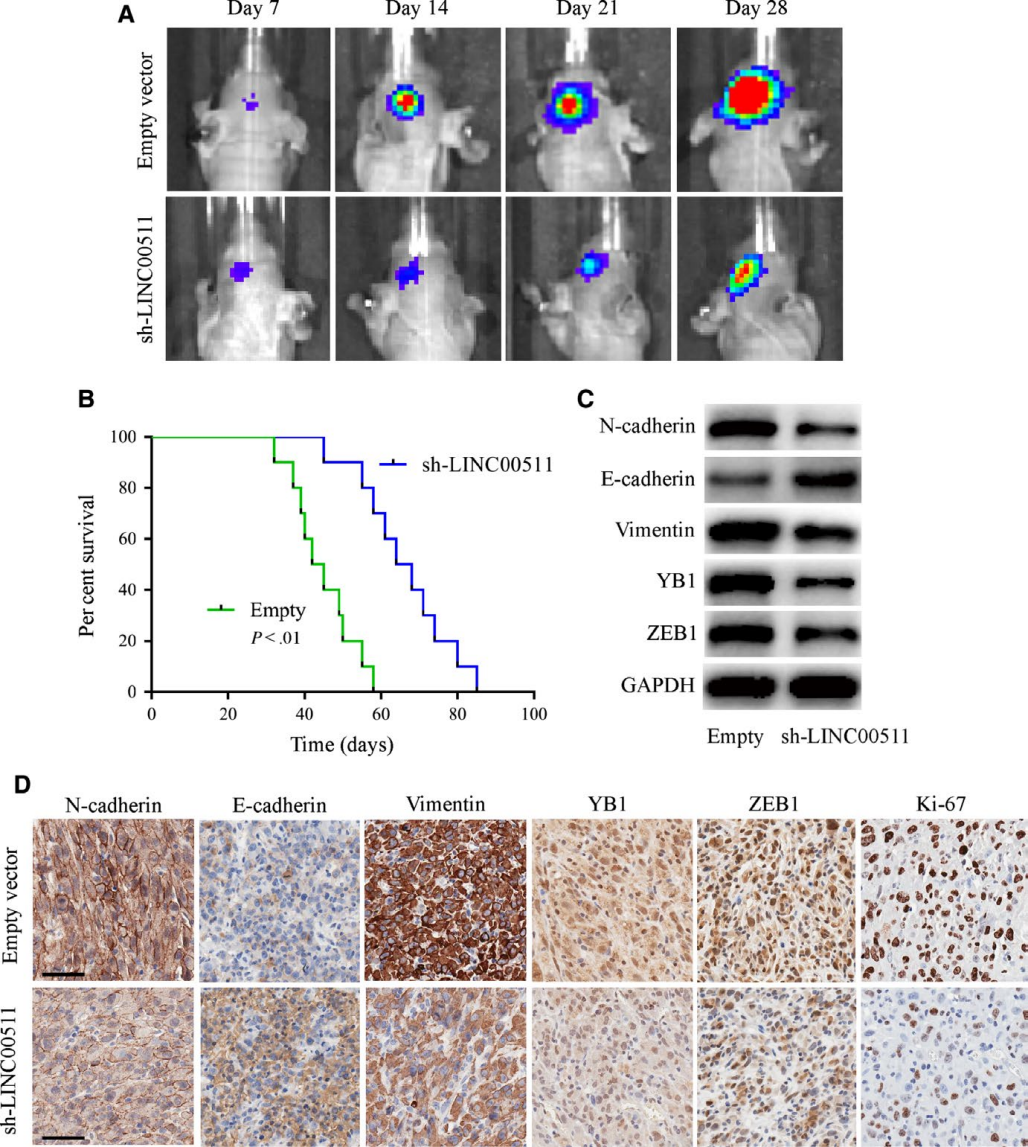
LINC00511 accelerates tumorigenesis of GBM cells in vivo. A, Representative bioluminescence images of orthotopic xenograft tumours transfected with empty vector or sh‐LINC00511 U251 cells and then injected into nude mice (10 mice per group). B, Survival curves of sh‐LINC00511 or empty vector transfected nude mice. C and D, Western blot and IHC assays were performed to investigate the changes of EMT‐related proteins in intracranial tumours. GAPDH acts as a loading control. Scale bar, 50 μm

The effect of LLINC00511 in vivo was evaluated using luciferase‐labelled U251 orthotopic xenograft models and analysed by bioluminescence imaging. We found that tumours from the sh‐LINC00511 U251 group were much smaller than the empty vector group (Figure 7A), and survival curves showed that sh‐LINC00511‐transfected xenografts revealed a better survival compared with the control (Figure 7B). Additionally, we performed Western blot and IHC on xenograft tumour tissues. xenografts inoculated with sh‐LINC00511 cells exhibited a lower malignant level with Ki‐67 reduction, and that EMT‐related proteins excluding E‐cadherin were significantly decreased compared with the empty vector group (Figure 7C,D). These results confirm that depleting LINC00511 inhibited GBM tumorigenesis in vivo.

## 4 DISCUSSION

Despite the rapid development of advanced technologies in clinical oncology, complete resection of GBM is still impossible because of its invasiveness.^9^ It has been reported that EMT in GBM is the key factor for its invasion and malignant behaviours^7^; however, the mechanism behind this activity remains largely unknown. Here, we investigated LINC00511, which may perform a pivotal role in EMT and induce invasion and malignant behaviours in GBM cells. Mechanistically, we found that ZEB1 positively regulates LINC00511. Meanwhile, LINC00511 sponges miR‐524‐5p, acting as a ceRNA to regulate YB1 expression, which can increase ZEB1 expression by binding to its promoter region. ZEB1 has been recognized as a key EMT transcriptional inducer. These data indicate that LINC00511/miR‐524‐5p/YB1/ZEB1 forms a positive feedback loop that could indicate the progression of GBM.

LINC00511, a non‐coding RNA of more than 200 bp, has been demonstrated to have vital functions in the proliferation and migration of human tumours.^26^, ^27^, ^28^, ^38^, ^39^ However, there is still little evidence defining the role of LINC00511 in EMT. In this study, we first detected the expression of LINC00511 was overexpressed in GBM tissues and cells. Confirming previous studies, we found that LINC00511 promoted GBM proliferation both in vitro and in vivo.^26^, ^27^, ^28^ Subsequently, we found that EMT‐related proteins, such as the mesenchymal markers Vimentin and N‐cadherin, were reduced, whereas the epithelial marker E‐cadherin was increased when LINC00511 was inhibited. The opposite results were found after enhancing LINC00511 expression. Wound healing, 3D spheroid invasion and transwell assays were performed to detect changes in the migration and invasion of GBM cells. The results further demonstrated that LINC00511 increased migration and invasion, and was beneficial to proliferation *via* enhancing EMT in GBM cells.

LncRNAs serve as guides, signals or decoys to effect target gene expression at transcriptional or post‐transcriptional levels.^40^ Emerging reports have revealed that cytoplasmic RNAs can act as ceRNAs, which was first proposed in 2011,^41^ to regulate miRNAs *via* binding and titrating them off their binding sites on protein‐coding messages, thus playing a crucial role in ceRNA networks in human cancers.^33^, ^42^, ^43^ For example, LINC00511 promotes tumorigenesis and stemness in breast cancer by interacting with miR‐185‐3p.^25^ In addition, LINC00511 promotes angiogenesis in pancreatic ductal adenocarcinoma by sponging miR‐29b‐3p.^26^ In our study, FISH and subcellular fractionation both revealed that LINC00511 was largely located in the cytoplasm rather than in the nucleus. Then, bioinformatics analysis, RIP and luciferase assays were used to verify that miR‐524‐5p can act as a novel and direct target for LINC00511.

In glioma, miR‐524‐5p has been demonstrated to have reduced expression compared with normal brain cells, suggesting it acts as a suppressor gene; miR‐524‐5p overexpression inhibited proliferation, migration, cell cycle progression and chemoresistance in GBM.^44^, ^45^, ^46^ However, the function of miR‐524‐5p in EMT has not been evaluated. Thus, we explored the exact function of miR‐524‐5p in GBM. In our study, we found that the EMT‐related markers Vimentin and N‐cadherin were up‐regulated, whereas E‐cadherin was down‐regulated in N3 and U251 cells transfected with miR‐524‐5p‐inhibitor. These results were rescued by co‐transfection with si‐LINC00511. Moreover, we obtained the target gene YB1 for miR‐524‐5p using bioinformatics databases.

YB1 is a member of the DNA/RNA‐binding protein family and has a common nucleic acid‐binding domain at its promoter region (CCAAT‐box), which possesses a high consensus sequence in eukaryotes.^37^, ^47^ Studies have reported that YB1 can regulate proliferation and metastasis by binding to lncRNAs^37^, ^47^ or miRNAs,^48^ and even demonstrate that YB1 effects EMT.^37^, ^49^ However, the relationship between LINC00511 and YB1 was not clear. Then, we further demonstrated that LINC00511 acts as a ceRNA of miR‐524‐5p to indirectly regulate YB1.

It has been reported that YB1 acts as a transcriptional inducer that enhances ZEB1 expression in pancreatic cancer.^37^ Thus, we wondered whether YB1 had the same effect on ZEB1 in GBM. ChIP and luciferase assays confirmed our original hypothesis. Western blot and qRT‐PCR analysis further demonstrated the same results.

As previously reported, the differential expression of lncRNAs is determined by upstream transcription factors.^31^ ZEB1 acts as a classical transcriptional inducer of EMT in many cancers.^13^, ^14^, ^37^ In this study, we found that ZEB1 could act as a transcription factor that binds LINC00511 in its promoter region to positively induce its expression, which completes the LINC00511/miR‐524‐5p/YB1/ZEB1 positive feedback loop.

## 5 CONCLUSION

We first identified a LINC00511/miR‐524‐5p/YB1/ZEB1 positive feedback loop in GBM cells, which promoted the migration and invasion of GBM cells by enhancing EMT. Therefore, we believe that LINCOO511 may be a potential therapeutic target for patients with GBM.

## CONFLICT OF INTEREST

The authors declare that they have no competing interests.

## AUTHORS' CONTRIBUTIONS

MP and JJ provided the direction of the experiments and guided the whole project. XD and YT mainly performed the experiments and wrote the paper. SL and PZ assisted the experiments. ZB was responsible for the analysis and interpretation of the data, and CL and JL were responsible for the collection of clinicopathological data.

## Data Availability

All data generated or analysed in the current study are included either in this article or in the additional files.
