# Supplementary material for: LINC00511 contributes to glioblastoma tumorigenesis and epithelial‐mesenchymal transition via LINC00511/miR‐524‐5p/YB1/ZEB1 positive feedback loop
Source: J Cell Mol Med. 2019 Dec 19;24(2):1474–87. doi: 10.1111/jcmm.14829 (PMC6991637; doi:10.1111/jcmm.14829)
Supplement: Supplementary file 1 [file JCMM-24-1474-s001.doc]

**Supplemental information**

**Figure S1**

**
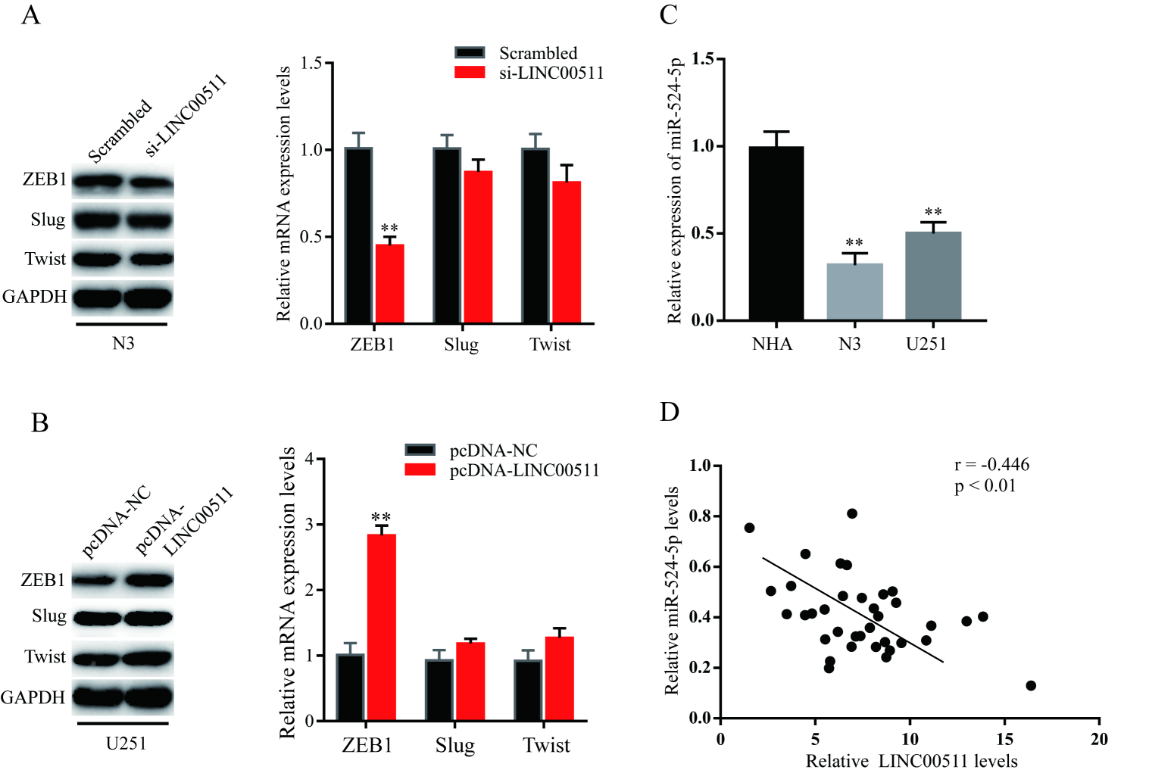
**

**Figure S1.**  A. The expression of EMT inducers in N3 cells transfected with si-LINC00511 at protein and mRNA levels. B. The expression of EMT inducers in U251 cells transfected with pcDNA-LINC00511 at protein and mRNA levels. C. MiR-524-5p expression in NHA, N3 and U251 cells were analyzed by qRT-PCR. D. Pearson's correlation method was used to demonstrate the relationship between LINC00511 and miR-524-5p. The data represent the mean of three independent experiments ± SEM. **P* < 0.05, ***P* < 0.01.

**Figure S2**


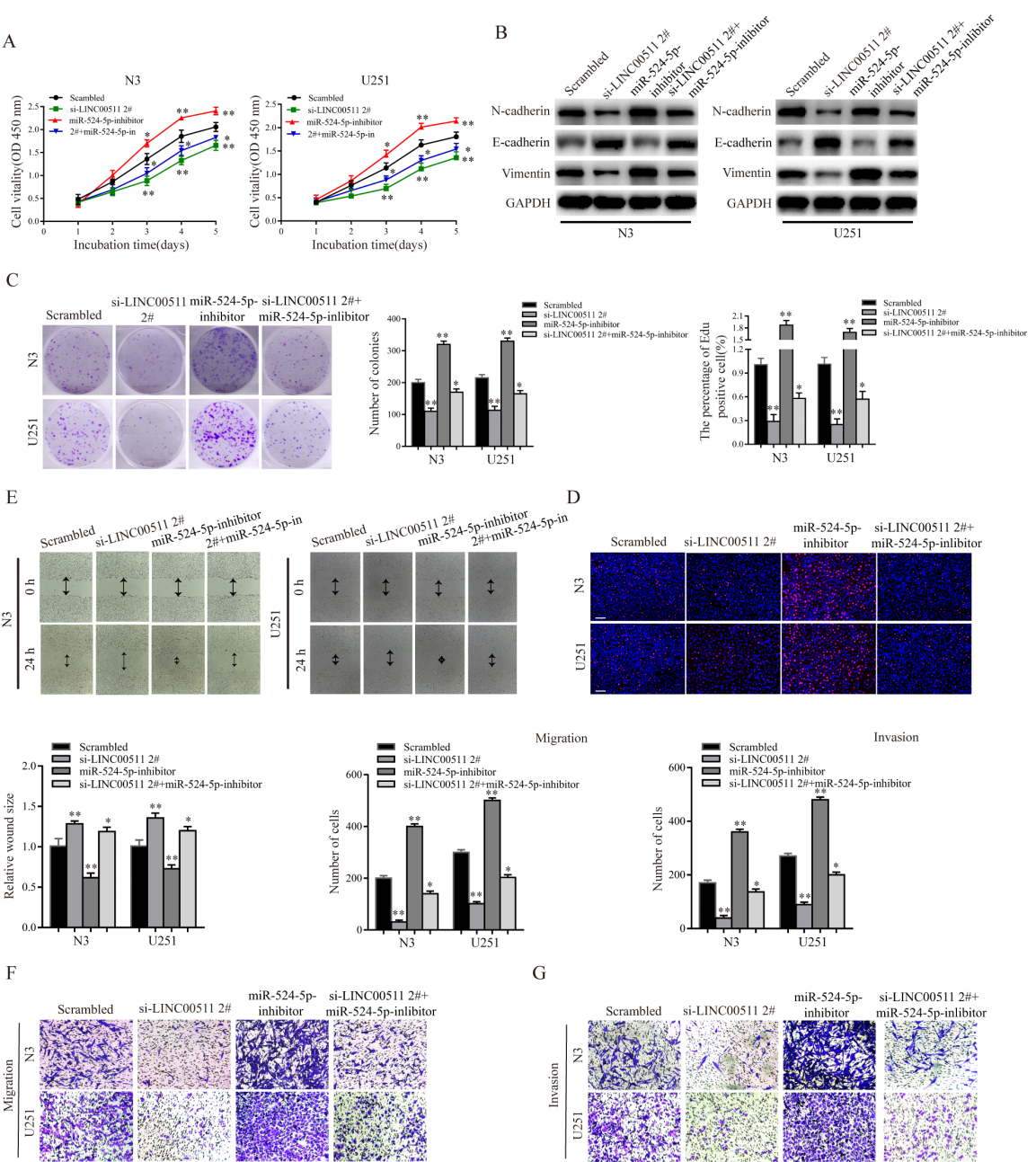


**Figure S2.** Effect of miR-524-5p to LINC00511 on GBM cells on EMT, proliferation, migration, and invasion. A. CCK8 assay was performed to determine the cell viability of si-LINC00511 and miR-524-5p inhibitor cotransfected N3 and U251 cells. B. EMT-induced ability for N3 and U251 cells after co-transfection with siLINC00511 and miR-524-5p inhibitor was detected by western blot assay. C and D. The proliferation ability of si-LINC00511 and miR-524-5p inhibitor co-transfected N3 and U251 cells was determined by colony formation and EdU assays. Scale bar, 100 μm. E-G. Wound healing and transwell assays were performed to display the migration and invasion ability in N3 and U251 cells after co-transfection with si-LINC00511 and miR-524-5p inhibitor. Values represent the mean ± SEM in three independent experiments. *P < 0.05, **P

< 0.01.

**Figure S3**


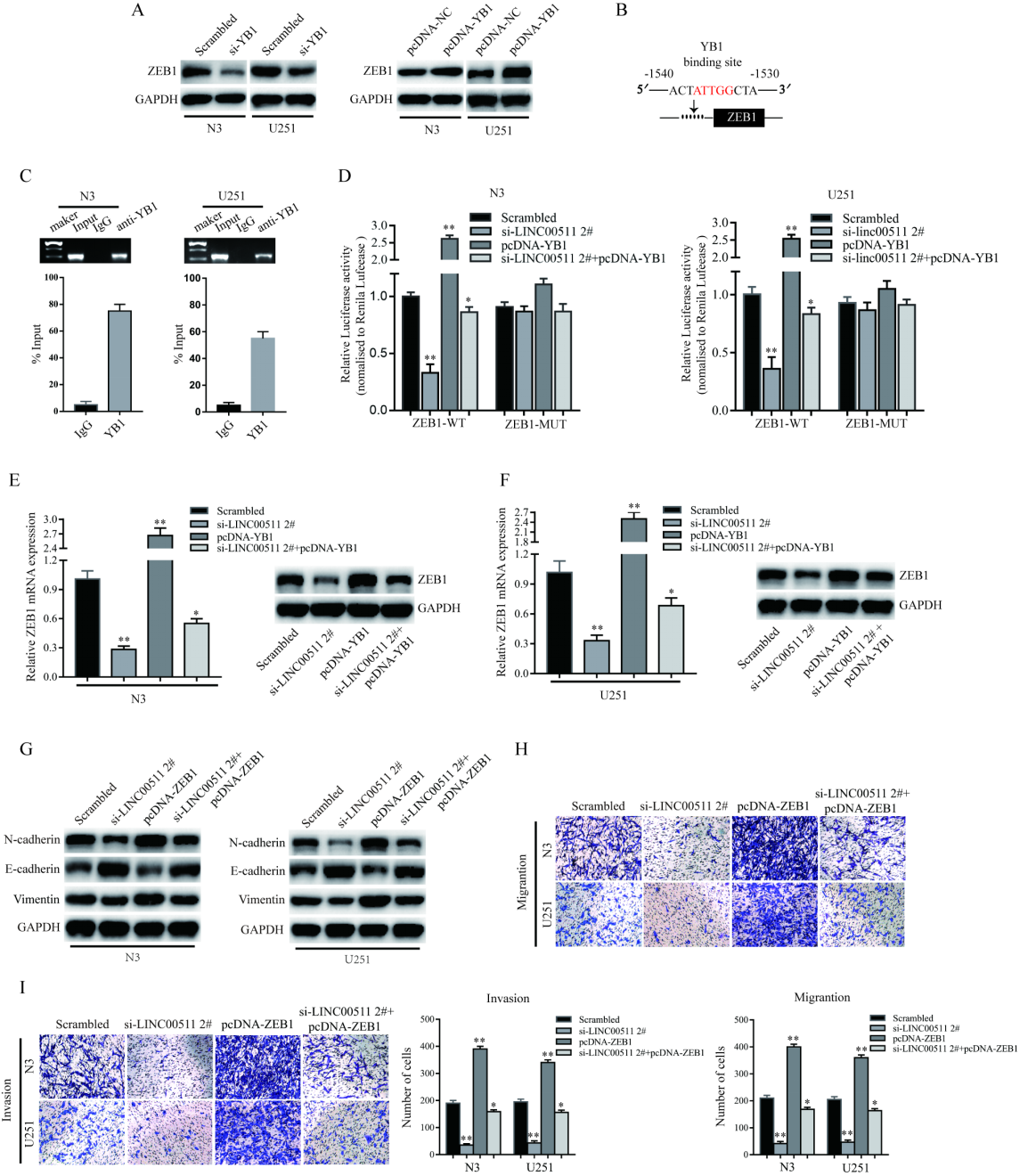


**Figure S3. LINC00511 indirectly promotes ZEB1 expression by sponging miR-524-5p to target YB1.** A. The expression of ZEB1 in N3 and U251 cells were analyzed by western blot assay. B. Schematic diagram of ZEB1 promoter region and YB1 potential binding site. C. ChIP assay was used to demonstrate the binding between YB1 and ZEB1 promoter region in N3 and U251 cells. D. The changes of luciferase activity in GBM cells co-transfected wild type or mutated ZEB1 promoter with si-LINC00511 and pcDNA-YB1. E and F. The expression of ZEB1 in si-LINC00511 and pcDNA-YB1 co-transfected N3 and U251 cells. G. The expression of EMT-related proteins in N3 and U251 cells. H and I. Transwell assay was performed to detect the co-transfected cells ability in migration and invasion. The data represent the mean of three independent experiments ± SEM. **P* < 0.05, ***P* < 0.01.

**Table S1.** Correlations between LINC00511 expression and clinicopathological indexes of GBM patients

| Indexes | Total (n) | LINC00324 | | P  Chi-squared  test P-value |
| --- | --- | --- | --- | --- |
|  |  | High No.Cases(18) | Low No.Cases(18) |  |
| Gender |  |  |  | 0.157 |
| Male | 24 | 10 | 14 |  |
| Female | 12 | 8 | 4 |  |
| Age |  |  |  | 0.700 |
| ＜50 | 9 | 5 | 4 |  |
| ≥50 | 27 | 13 | 14 |  |
| Tumor location |  |  |  | 0.180 |
| Frontal | 16 | 10 | 6 |  |
| Non-frontal | 20 | 8 | 12 |  |
| Tumor size |  |  |  | 0.026* |
| ≤3CM | 8 | 2 | 8 |  |
| >3CM | 28 | 16 | 10 |  |
| Status |  |  |  | 0.001* |
| Survival | 16 | 3 | 13 |  |
| Death | 20 | 15 | 5 |  |
| recurrence |  |  |  | 0.011* |
| YES | 11 | 9 | 2 |  |
| NO | 25 | 9 | 16 |  |
| IDH1/2 genotype |  |  |  | 0.043* |
| Mutation | 7 | 1 | 6 |  |
| Wild-type | 29 | 17 | 13 |  |
| MGMT  promoter status |  |  |  | 0.248 |
| Methylated | 11 | 3 | 6 |  |
| Unmethylated | 25 | 15 | 12 |  |

Abbreviations: IDH1/2, Isocitrate dehydrogenase 1 and 2;

MGMT, O-6-methylguanine-DNA-methyltransferase;

P < 0.05 was considered significant.

**Table S2.** Primers used for qRT-PCR

| Primers | Forward-primer (5’-3’) | Reverse-primer(5’-3’) |
| --- | --- | --- |
| LINC00511 | CTGCTTGGGTGTGGGACTGA | CGATGAGGTCATACGCCGTAA |
| ZEB1 | CGCAGTCTGGGTGTAATCGTAA | GACTGCCTGGTGATGCTGAAA |
| Slug | TGCGATGCCCAGTCTAGAAA | GTGTCCTTGAAGCAACCAGG |
| Twist | CTAGAGTCTGAGATGCCCCG | AGTTCTGGGAGACACATCGG |
| miR-524-5p | GCCCGCTACAAAGGGAAGCAC | ATCCAGTGCAGGGTCCGAGG |
| GAPDH | GAACGGGAAGCTCACTGG | GCCTGCTTCACCACCTTCT |
| U6 | CTCGCTTCGGCAGCACA | AACGCTTCACGAATTTGCGT |

**Table S3.** Sequences used for siRNAs

| Name | sequence |
| --- | --- |
| si-LINC00511 1# | CCAAGUUAGCCUCUCCCUU |
| si-LINC00511 2# | CCAUCGAUCGACCUACAAA |
| si-LINC00511 3# | GCUUGUGCCCUUGGAAUUA |
| si-ZEB1 | GGAAGAGGAGGAGGATAAA |
| si-YB1 | GCAGACCGUAACCAUUAUATT |

**Table S4.** Primers used for CHIP

| Gene | Binding site | Forward-primer (5’-3’) | Reverse-primer(5’-3’) |
| --- | --- | --- | --- |
| LINC00511-TFBS | Site 1 | ATAGGAAGCCCTTGGTTTGG | CAGGATGGTCTCGATCCACT |
|  | Site 2 | ACTCTACCAGACGGGGCTTT | CACGAAACTGAAAGCTGCTG |
|  | Site 3 | GTGGCGCCGGGGGCAGGTCC | GCGTGCAAAGCCCCGGCACC |
| ZEB1-TFBS | Site 1 | TAAGGACTAAAGGGATGA | ACGTAGCCAATAGTAGATG |
